# Tuberculosis, Fiji, 2002–2013

**DOI:** 10.3201/eid2203.151903

**Published:** 2016-03

**Authors:** Lorenzo Pezzoli, Shakti Gounder, Talatoka Tamani, Mary Raori Daulako, Frank Underwood, Sakiusa Mainawalala, Vasiti Nawadra-Taylor, Eric Rafai, Laura Gillini

**Affiliations:** Epidemiology Consultant, Suva, Fiji (L. Pezzoli);; Ministry of Health, Suva (S. Gounder, T. Tamani, M.R. Daulako, F. Underwood, S. Mainawalala, E. Rafai, V. Nawadra-Taylor);; World Health Organization, Suva (L. Gillini)

**Keywords:** Tuberculosis and other mycobacteria, Fiji, control program, epidemiology, tuberculosis, South Pacific, trends, bacteria

## Abstract

During 2002–2013, a total of 1,890 tuberculosis cases were recorded in Fiji. Notification rates per 100,000 population increased from 17.4 cases in 2002 to 28.4 in 2013. Older persons were most affected, but tuberculosis also increased sharply in persons 25–44 years of age.

Tuberculosis (TB) remains a major cause of illness and death globally (*1*,*2*). Fiji, which comprises 332 islands and a total land area of 18,333 km^2^, is located in the center of the South Pacific. In 2013, Fiji’s Ministry of Health estimated a population of 882,860 persons. The public health system is organized into 4 divisions, 20 subdivisions, and 80 medical areas. The National Tuberculosis Programme (NTP) was established in 1951 and in 1997 adopted the directly observed treatment strategy (DOTS). The 3 DOTS centers are P.J. Twomey, covering Central and Eastern Divisions; Labasa, covering Northern Division; and Lautoka, covering Western Division.

In 2012, per 100,000 population, reported TB incidence in Fiji was 24 (95% CI 21–27) cases, prevalence was 30 (95% CI 10–61), and the case-fatality rate was 1.7 (*3*,*4*). To assess the status of TB epidemiology in Fiji and identify areas of intervention, we used surveillance data to retrospectively analyze trends in TB cases reported by the NTP from 2002 through 2013. 

## The Study

Case notification data for 2002–2013 were obtained from the 3 NTP TB registers (1 per DOTS center). Data on geographic location (up to medical area level) of TB cases were available electronically from 2005 and on age groups from 2010.

We defined a TB case based on >1 of the following diagnostic criteria: sputum or body fluid and tissue that was smear-positive for acid-fast bacilli (AFB), culture-positive for *Mycobacterium tuberculosis* complex, or both; or clinical appearance, radiographic appearance, or both consistent with TB. Cases caused by documented nontuberculous mycobacteria were excluded. We defined a smear-positive pulmonary TB (PTB+) case as >2 initial sputum smear examinations (direct smear microscopy) AFB-positive; or 1 sputum examination AFB-positive plus radiographic abnormalities, symptoms, or both consistent with active pulmonary TB. We defined a smear-negative pulmonary TB (PTB–) case as symptoms suggestive of TB, with >3 initial smear examinations negative for AFB but with no response to a treatment course with broad-spectrum antimicrobial drugs (excluding quinolones), and/or with radiologic abnormalities consistent with pulmonary TB, followed by a clinician’s decision to treat for TB. We defined an extrapulmonary TB (EPTB) case as TB only of organs other than the lungs (e.g., pleura, lymph nodes, abdomen, genitourinary tract, skin, joints and bones, meninges), diagnosed on the basis of 1 culture-positive specimen, or histologic or strong clinical evidence consistent with active extrapulmonary disease, followed by a clinician’s decision to treat with a full course of anti-TB chemotherapy.

We described cases by type, patient age, and location. Case notification rates (CNRs) were measured respectively as TB cases or deaths during a given year divided by the population estimate for that year. Age-standard CNRs were calculated by dividing the number of TB cases in each age group by the total number of population estimated in that age group. We analyzed TB case notifications for trends using simple linear regression models.

During 2002–2013, a total of 1,890 new cases of TB were reported. Since 2002, the only significant linear upward trends were for all cases (coefficient 7.86, p = 0.046) and PTB+ cases (coefficient 4.02, p = 0.004). Since 2007, the model showed significant linear trends for all cases (coefficient 26.82, p<0.001), PTB+ cases (coefficient 9.00, p = 0.002), PTB– cases (coefficient 12.14, p = 0.001), and EPTB cases (coefficient 5.68, p = 0.037); the proportion of PTB– cases (coefficient 0.04, p = 0.004); and the CNR (coefficient 2.97, p<0.001).

In Fiji’s 4 divisions during the study period, the highest average CNR was reported from the Central Division (22.5 cases/100,000 population) and the lowest from the Eastern Division (10.9 cases/100,000 population) (Figure; Table 1). The 25–34-year age group had the most cases (198 [22%]); the fewest cases were reported in the 0–4-year group (28 [3%]). The CNR for persons >55 years of age consistently exceeded 40 cases/100,000 population. In 2013, the CNR for persons >65 years of age increased to 75.7 cases/100,000 population. In persons 25–34 years of age, the CNR increased from 25.8 cases/100,000 population in 2012 to 42.2 cases/100,000 population in 2013 (Table 2).

**Figure:**
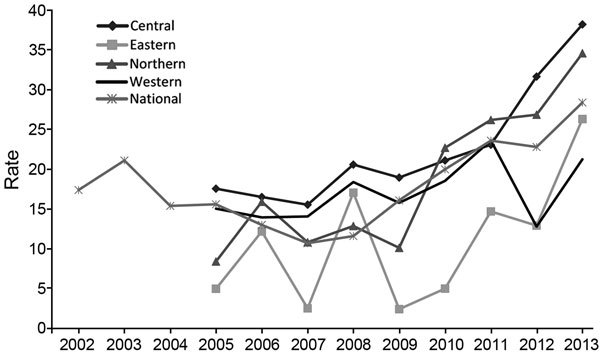
National tuberculosis case notification rates (2002–2013) and by division (2005–2013), Fiji. Rates are number of cases per 100,000 population.

**Table 1:** New tuberculosis cases, by year and type, Fiji, 2002–2012*

Year	No. (%)			Total no., N = 1,890
	EPTB	PTB–	PTB+	
2002	38 (25.3)	38 (25.3)	74 (49.3)	150
2003	53 (29.6)	48 (26.8)	78 (43.6)	179
2004	31 (23.7)	38 (29.0)	62 (47.3)	131
2005	40 (30.3)	29 (22.0)	63 (47.7)	132
2006	18 (15.9)	22 (19.5)	73 (64.6)	113
2007	34 (36.6)	7 (7.5)	52 (55.9)	93
2008	19 (18.6)	5 (4.9)	78 (76.5)	102
2009	38 (26.8)	21 (14.8)	83 (58.5)	142
2010	45 (25.1)	45 (25.1)	89 (49.7)	179
2011	44 (20.7)	62 (29.1)	107 (50.2)	213
2012	40 (19.5)	54 (26.3)	111 (54.1)	205
2013	71 (28.3)	74 (29.5)	106 (42.2)	251

*EPTB, extrapulmonary tuberculosis; PTB, pulmonary tuberculosis; –, smear negative; +, smear positive.

**Table 2:** Tuberculosis cases, by age group and CNR, Fiji, 2002–2012*

Age group, y	No. (CNR)				Overall, no. (mean CNR)
	2010	2011	2012	2013	
0–4	4 (4.5)	2 (2.3)	11 (12.4)	11 (12.6)	28 (7.9)
5–14	16 (9.4)	12 (7.0)	10 (5.8)	15 (8.9)	53 (7.7)
15–24	29 (17.0)	44 (25.6)	42 (24.5)	47 (27.9)	162 (23.7)
25–34	44 (30.1)	55 (37.3)	38 (25.8)	61 (**42.2**)	198 (33.9)
35–44	27 (22.4)	30 (24.7)	28 (23.1)	45 (37.8)	130 (27.0)
45–54	22 (22.8)	27 (27.8)	34 (35.0)	33 (34.7)	116 (30.0)
55–64	31 (**52.6**)	25 (**42.0**)	26 (**43.8**)	26 (**44.6**)	108 (**45.7**)
>65	18 (**43.5**)	18 (**43.1**)	29 (**69.5**)	31 (**75.7**)	96 (**58.0**)
All	191 (21.4)	213 (23.6)	218 (24.2)	269 (30.5)	891 (24.9)

*Bold type indicates CNR >40 cases/100,000 population. CNR, case notification rate.

## Conclusions

Our comprehensive evaluation of TB trends in Fiji shows a steady increase in CNRs since 2007. Older age groups were disproportionally affected. This finding is not surprising because the elderly strata of the population are more likely to have been infected and are more prone to reactivation of dormant mycobacteria (*5*). TB in these persons is of concern because they are more likely to die or have poorer outcomes because of old age and concurrent conditions. The sudden increase in CNR in the 25–34 and 35–44 age groups during 2012–2013 is also of major concern. This finding might indicate increased transmission within the community because most cases in these younger adults are presumably due to recent infections.

The relative decrease in PTB+ cases below the programmatic aim of 50% smear-positive cases contrasts with results from a previous analysis (*4*) and reflects a concurrent increase in PTB– cases diagnosed rather than an increase in EPTB cases. The observation of increased PTB– cases might imply greater awareness and sensitivity by medical practitioners to diagnosing clinical TB syndromes or lower specificity in the diagnosis (i.e., a proportion of PTB– cases might be attributable to other diseases). The 106 PTB+ cases detected in 2013 remain a relatively high number because these patients are more infectious and should be detected as early as possible.

Our assessment is subject to several limitations. First, this is a review of programmatic data, so it is not possible to know whether the trends represent a change in the disease incidence or a change in programmatic functioning. Second, TB data were collated at different sources and were mostly paper based and not easily accessible. Finally, although some major hospitals (e.g., private practices or the Colonial War Memorial Hospital, Suva, Fiji) participate in active case detection, they are not completely involved in TB treatment of patients and might underreport cases.

The increase in CNRs during the study period can be attributed to several factors. First, during 2008–2011, program reviews led to a strengthened TB surveillance system (*6*). The increased CNR also might be attributed to enhanced program support by the Global Fund. Fiji first received money from the Global Fund to Fight AIDS, Tuberculosis and Malaria (http://www.theglobalfund.org) for TB in 2010 and had received US $7,570,339 through 2013. These enhanced program activities are likely to have contributed to the increase in notifications in recent years. However, they cannot fully explain the rise in CNRs. In the 2014 Global Tuberculosis Report, the World Health Organization estimated TB incidence in Fiji at 37 (95% CI 33–42) cases/100,000 population in 2013, which is a considerable increase over the 24 (95% CI 21–27) cases/100,000 population in 2012 (*1*,*7*) and suggests that the program still needs to optimize its case-finding potential. Prevention and control activities should be intensified in younger adults to reduce the number of new infections. Finally, analyzing trends is only 1 aspect of the evaluation of DOTS, and further research on measuring outcomes should be explored (*8*).
